# The Early Emotional Bond: An Evolutionary-Developmental Perspective Integrating Psychoanalysis, Neuroscience, and Cross-Cultural Evidence

**DOI:** 10.3390/brainsci16040355

**Published:** 2026-03-26

**Authors:** Maria Cafaro, Laura Ambrosecchia, Valeria Cioffi, Enrica Tortora, Raffaele Sperandeo, Daniela Cantone

**Affiliations:** 1 Italian Association of Psychoanalytic Psychotherapy for Children, Adolescents, and Families (AIPPI), 80131 Napoli, Italy; marinacafaro5@gmail.com (M.C.); lauraambro84@gmail.com (L.A.); 2 SiPGI–Postgraduate School of Integrated Gestalt Psychotherapy, 80058 Torre Annunziata, Italy; enricatortora1604@gmail.com; 3 Department of Neuroscience, Reproductive and Odontostomatological Sciences, University of Naples Federico II, 80131 Naples, Italy; raffaele.sperandeo@gmail.com; 4 Dipartimento di Psicologia, Università degli Studi della Campania Luigi Vanvitelli, 81100 Caserta, Italy; daniela.cantone@unicampania.it

**Keywords:** attachment, early, relationships, psychoanalysis, developmental neuroscience, epigenetics, brain plasticity, emotion regulation

## Abstract

**Highlights:**

**What are the main findings?**

- Early attachment emerges from the dynamic interaction of biological, neural, relational, and cultural factors rather than from a single universal developmental pathway.
- Genetic and epigenetic variability creates windows of differential susceptibility, making early relational experiences key modulators of neurodevelopmental and emotional outcomes.

**What are the implications of the main findings?**

- Early attachment vulnerabilities should be understood as context-dependent adaptive strategies rather than fixed risk factors for psychopathology.
- Preventive and clinical interventions are most effective when they adopt an integrated, culturally sensitive approach that addresses multiple developmental domains simultaneously.

**Abstract:**

**Background/Objectives**: This article is a narrative review that examines the development of attachment from intrauterine life to the first thousand days of a child’s life, integrating psychoanalytic, neuroscientific, genetic, and cross-cultural perspectives. Biological, relational, neurological, and cultural factors interact and shape individual differences in socio-emotional functioning. This paper aims to propose a reinterpretation of early attachment, describing it as both a clinical and relational phenomenon and an adaptive process inscribed in human evolutionary history, according to the Four-Domain Integrative Framework described herein. **Methods**: The review examined three main areas of evidence: early attachment characteristics, cross-cultural caregiving variations, and genetic and epigenetic mechanisms underlying environmental sensitivity. **Results**: The review first identified seven characteristics of early attachment (proximity seeking, emotional attunement, intrauterine experiences, maternal holding, security patterns, brain plasticity, and maternal stress) which represent developmental mechanisms that generate individual differences in trust, self-regulation, resilience, and psychopathological vulnerability. Second, cross-cultural variations in six distinct caregiving contexts were examined, demonstrating that secure attachment emerges through culturally specific pathways, differentially influencing motor development, sleep patterns, hypothalamic–pituitary–adrenal axis maturation, and social skills. Finally, the differential susceptibility model was provided through the analysis of five genetic and epigenetic systems (oxytocin receptor gene, serotonin transporter gene, dopamine receptor gene, glucocorticoid receptor methylation, and fetal programming) that modulate environmental sensitivity. **Conclusions**: Biological, relational, neurological, and cultural factors interact and shape individual differences in socio-emotional functioning.

## 1. Introduction

Early emotional bonding is one of the key factors in human development. The relational experiences of the first thousand days of life, from gestation to early childhood, form the foundation on which the mind, the self, and the ability to relate to others are built. Psychoanalysts have always emphasized the importance of the primary relationship, from Bowlby’s attachment theories to Winnicott’s reflections on the role of the mother-environment. In recent decades, however, affective neuroscience has provided a solid basis for psychoanalytic theories by showing how interpersonal experiences in early childhood directly influence brain development, emotional regulation, and psychological resilience [1]. We will analyze some of the contributions from these disciplines, bringing together theoretical models and empirical data to understand how early emotional bonding, as a biological, psychological, and relational matrix, underlies human development. In this sense, intrauterine life has become a subject of fascination and reflection for those who study the birth and development of the human mind.

Freud [2] had already hypothesized that “There is much more continuity between intrauterine life and early childhood than the impressive break of the act of birth would have us believe” (p. 286). Following this profound reflection, we can think about the continuum that characterizes life before and after birth and the impact, in evolutionary terms, that this prenatal space has on the future of the individual. Perinatal psychology invites us to reconsider the origin of individual developmental trajectories. Personal narratives do not begin at birth but are already rooted in the prenatal stages of development. Early relational and somatic experiences constitute a pre-history that shapes the emotional and psychological future of the individual.

Contrary to the traditional view that considered the fetus a passive organism, the most recent research shows that as early as the sixteenth week of gestation, the fetus exhibits exploratory behaviors, sensory responses, and implicit learning abilities. Intrauterine experiences—such as the tone of the mother’s voice, heart rate, and hormonal changes—are stimuli that the fetus perceives and integrates, contributing to the formation of bodily and emotional memory. Alessandra Piontelli’s pioneering studies [3] have documented a significant continuity between fetal behavioral characteristics and postnatal temperament. Her work has highlighted how individual differences predictive of subsequent development already emerge in the prenatal period. The research of psychoanalysts such as Negri and Piontelli [4], applied to the study of fetal behavior using ultrasound investigation techniques, has shown that the fetus is immersed in a relational environment that stimulates and shapes it, anticipating the construction of the bodily and affective self. From a psychoanalytic standpoint, it is an implicit dialog that the mother, together with her environment, builds with the fetus in utero. Even before birth, the child is involved in an intersubjective relationship with the mother, mediated by neurophysiological and affective signals.

The impact of the mother, as the one who contains, and of the environment in which the woman is placed, as a space that in turn contains the mother–child dyad, is fundamental for the construction of the fetus’s mind, which is actively involved in this work of interaction and construction of a very early intersubjectivity. Neuroscientific studies [5] show, for example, that maternal stress, depression, and traumatic experiences during pregnancy are associated with alterations in the brain connectivity of the fetus, particularly in the amygdala and the hypothalamic–pituitary–adrenal axis, and may predispose the child to difficulties in emotional regulation and stress response.

Prenatal sensory and motor experiences are stored in implicit memory, a predominantly emotional system that matures in the last trimester of pregnancy and involves the amygdala, basal ganglia, cerebellum, and cortical areas of the right hemisphere [6,7,8]. Neural connectivity develops progressively from the twenty fourth week, with a predominance of the right hemisphere until the first two years of life [9,10,11]. From the thirtieth week, the main connections that constitute the neurobiological substrate for basic affective functions are established. Fetal brain maturation follows a caudal–rostral progression (from the brainstem to the cerebral cortex). Theoretically, by 28 weeks, a biological framework is created that supports the emergence of autonomous primary mental activity [12]. Alterations in this early connectivity are implicated in developmental disorders, from autism spectrum disorders to major depression. These neurological maturation processes continue after birth, requiring appropriate relational experiences for the normal development of the limbic nuclei. The right amygdala and anterior cingulate cortex are strongly shaped by early caregiving experiences. These structures play a primary role in the development and maintenance of emotional and social attachment. From an evolutionary perspective, early attachment represents an adaptive strategy aimed at survival: the search for proximity to the caregiver, the ability for emotional attunement, and the emergence of individual differences already in the prenatal phase allow the child to adapt functionally to the environment. These characteristics not only promote immediate survival but also constitute fundamental elements for the construction of personality, affective–relational regulation, and motivation in adulthood. To ensure clarity and consistency in the presentation, it is necessary to clarify the use of certain key terms which, although referring to related phenomena, are not perfectly interchangeable (Table 1). In this review, the term “attachment” is used in a broad developmental sense that includes its prenatal precursors, the neurobiological, sensory, and affective processes that, from the intrauterine period onward, lay the foundations for the postnatal mother–infant bond. As clarified in Table 1, where the term “early emotional bond” is used as an umbrella concept encompassing both attachment and bonding, this extended use of the term “attachment” to prenatal processes is theoretically grounded in perinatal psychology and in neuroscientific evidence on fetal learning and implicit memory [3,4,6,8,13].

### 1.1. The Four-Domain Integrative Framework: A Dynamic Model of Early Attachment

The development of early attachment is an extremely complex phenomenon that cannot be fully understood through single-perspective approaches. Different perspectives have analyzed this phenomenon in parallel, generating a fragmented understanding that constitutes a theoretical and practical limitation. Psychoanalytic theory has deepened our understanding of relational dynamics, yet has engaged less systematically with neurobiological mechanisms; neuroscience has illuminated the brain correlates of attachment, yet has insufficiently accounted for cultural variability in caregiving; genetic research has examined hereditary predispositions, yet without fully integrating relational and contextual factors; and cross-cultural studies have documented the diversity of caregiving practices, yet without articulating their biological and neural underpinnings. No existing model has proposed a systematic account of how these domains interact dynamically and bidirectionally across development—a gap that the present review aims to address. While psychoanalytic theory has emphasized the relational dynamics that characterize early attachment, neuroscience has focused primarily on the brain mechanisms involved in the development of this phenomenon. Genetics has examined the influence of hereditary predispositions on attachment, and cross-cultural research has revealed contextual variations.

In light of these considerations, we propose a Four-Domain Integrative Framework that explains the dynamic, bidirectional interactions between: (1) biology/genetics, (2) neuroscience, (3) relational experiences, and (4) cultural contexts.

**Figure 1:**
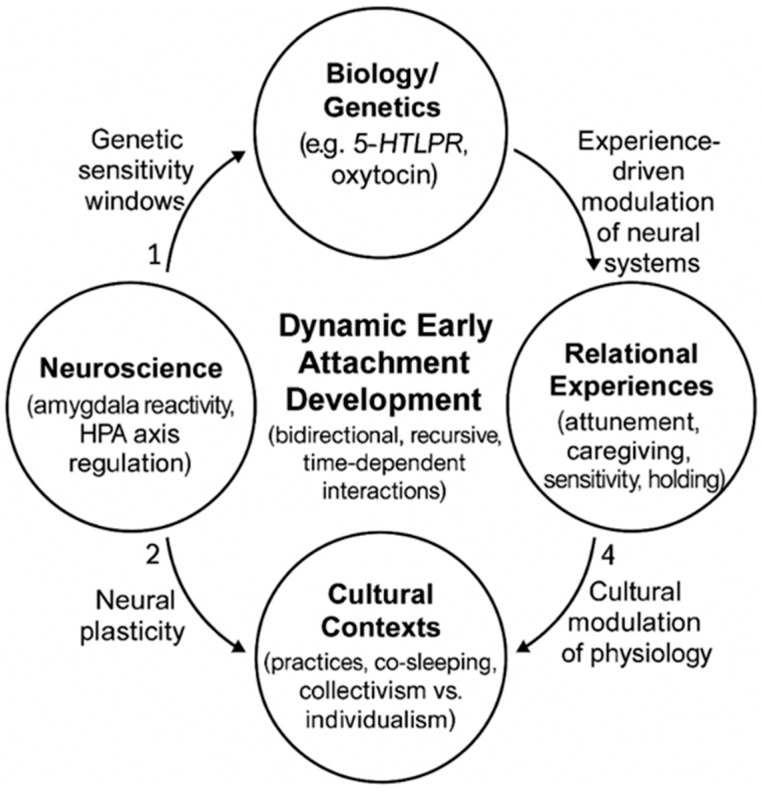
Four-Domain Integrative Framework of early attachment. The Four-Domain Integrative Framework describes early attachment as the emergent result of dynamic, bidirectional interactions among biology/genetics, neuroscience, relational experiences, and cultural contexts. Unlike traditional biopsychosocial models, this framework emphasizes (a) temporal cascades and differential susceptibility, (b) recursive causality across levels, and (c) evolutionary-adaptive plasticity. Genetic predispositions (e.g., 5-HTTLPR) shape periods of heightened neural plasticity; relational experiences such as maternal attunement modulate oxytocin and HPA-axis activity; cultural practices influence physiological regulation; and these domains mutually co-regulate each other across development.

The overall structure of the proposed Four-Domain Integrative Framework and the dynamic, bidirectional interactions among its components are illustrated in Figure 1.

Our integrative framework recognizes bidirectional causality at all levels. While traditional biopsychosocial models assume a unidirectional or additive relationship between different domains, our integrative framework emphasizes the properties that emerge from their interactions. Cultural practices (e.g., co-sleeping) do not simply reflect attachment patterns, but have been associated with modulation of physiological regulation, including potentially the HPA axis and cortisol rhythms [14], which may in turn influence the child’s ability to regulate affect, thereby reinforcing culturally adaptive behavioral patterns. Similarly, maternal attunement is closely linked to oxytocin activity: recent studies show that oxytocin reactivity in infants and mothers during interaction is associated with maternal sensitivity and the subsequent development of secure attachment [15,16], suggesting how relational experiences may recursively influence the biological substrate.

It integrates an evolutionary-adaptive perspective that reformulates vulnerability as context-dependent plasticity, rather than limiting itself to a dichotomous categorization of evolutionary outcomes in terms of health or pathology. From an evolutionary-adaptive perspective, what appears as “insecure attachment” in Western individualistic frameworks may represent adaptive strategies in collectivist or high-risk ecologies [17,18]. The differential susceptibility model [19] illustrates this principle: “orchid children” (carriers of sensitivity alleles) develop optimally in favorable environments but show greater vulnerability in adverse contexts, while “dandelion children” remain stable in all conditions. This genetic diversity represents an evolutionary hedging strategy that maintains the adaptability of the population.

#### Points of Mechanistic Integration

The framework operates through four main mechanisms:

1. Epigenetic Programming as Biological Memory of Relational Experience

The quality of maternal care modulates methylation patterns of the glucocorticoid receptor (NR3C1) during the prenatal and early postnatal periods [20,21], influencing the reactivity of the HPA axis. Interpreted within the integrative framework, these epigenetic changes constitute a biological “memory” of the relational context, which interacts with genetic predispositions and cultural context without rigidly determining outcomes [22,23].

Concrete examples of this mechanism emerge from recent studies. Research conducted during the COVID-19 pandemic showed that mothers and newborns exposed to lockdown during the first trimester of pregnancy had significantly lower levels of methylation of NR3C1 and SLC6A4 than those exposed in the second or third trimester, with effects persisting even after controlling for confounding variables [24]. This evidence suggests that specific time windows during gestation represent critical periods of epigenetic susceptibility to maternal stress. Furthermore, longitudinal studies have documented that increased methylation of NR3C1 in infants, associated with prenatal maternal depression, predicts increased cortisol responses to stress at 3 months of age [25]. These epigenetic patterns are not deterministic but plastic: research on animal models shows that high-quality maternal care can reduce Nr3c1 methylation even in the presence of previous adverse experiences [20,21], suggesting windows of opportunity for early interventions that may influence epigenetic programming.

2. Neural Plasticity as a Substrate of Relational Internalization

During the first years of life, the limbic and prefrontal circuits, together with the right hemisphere, show high plasticity and sensitivity to relational inputs [8,9,10]. The quality of caregiving and maternal attunement are associated with modulation of these neural networks and have been linked to improved regulation of emotions and the development of secure relational patterns [15,16]. Cultural differences in caregiving, such as the degree of physical contact and interactive synchrony, indirectly influence the development of structures such as the amygdala and insula, highlighting how relational experience and cultural context shape early neural plasticity [26].

Neural plasticity as a substrate for relational internalization is supported by specific evidence. Neuroimaging studies have revealed that mothers with greater responsive sensitivity show more coordinated activation patterns in the limbic and prefrontal regions during interaction with their children [27]. The role of oxytocin is particularly relevant: longitudinal research has shown that maternal sensitivity predicts secure infant attachment only when accompanied by an increase in maternal oxytocin during parent–child interaction, highlighting the bio-relational nature of this process [28]. Furthermore, low levels of basal maternal oxytocin at 3 months significantly predict emotional reactivity and withdrawal behaviors in the child at 3.5 years, even when controlling for maternal symptomatology and adult attachment style [29]. These correlational findings are consistent with the hypothesis that early relational experience, mediated by neurochemical systems such as oxytocin, becomes encoded in emerging brain structures, contributing to neural patterns that may persist over time.

3. Cultural Practices as Scaffolds for Gene Expression and Neural Development

Cross-cultural variations in caregiving represent evolutionary adaptations that shape development according to environmental specificities. For example, co-sleeping in collectivist cultures maintains mother–child physiological synchrony and may influence cortisol rhythms, indirectly contributing to adaptive emotional regulation strategies [14]. These practices interact with gene variants such as OXTR, influencing attachment outcomes [30,31]. The interaction between cultural practices and genetic expression is supported by specific cross-cultural evidence. A comparative study has shown that co-sleeping, prevalent in collectivist cultures, has been associated with maintained mother–child physiological synchrony and modulation of circadian cortisol rhythms, which may indirectly influence the development of adaptive emotional regulation strategies [14]. These cultural practices are not biologically neutral: they interact with genetic variants such as OXTR rs53576, where the G allele is associated with greater social sensitivity and secure attachment, but its phenotypic effects vary significantly depending on the cultural context of caregiving [32].

Furthermore, recent research on babywearing (continuously carrying the baby) has shown that prolonged physical contact increases the release of oxytocin in both parents and children, while simultaneously reducing cortisol levels [16,26]. This example illustrates how specific cultural practices act as “scaffolds” that simultaneously shape gene expression, neural development, and behavioral patterns, creating culturally specific but biologically rooted developmental trajectories.

4. Developmental Cascades: From the Molecular to the Behavioral Level

The framework describes how genetic, epigenetic, neural, relational, and cultural factors influence each other throughout development:

1. Genetic: For example, DRD4 variants confer greater environmental sensitivity [33].
2. Epigenetic: Maternal stress can modulate NR3C1, increasing stress reactivity [20,21].
3. Neural: The plasticity of limbic and prefrontal circuits makes the brain receptive to relational experience [15,16].
4. Relational: The quality of maternal attunement or the extended network modulates emotional regulation and attachment patterns.
5. Cultural: Cultural practices can amplify or attenuate the effects of other domains, promoting adaptations consistent with the environment [14,17,34].
6. Behavioral outcome: By early childhood, differences in emotional regulation, social cognition, and exploratory behavior emerge, reflecting the integration of all four domains.

A paradigmatic example of this integrated cascade emerges from prospective longitudinal studies. Children carrying the short 5-HTTLPR variant (genetic level), exposed to prenatal maternal stress that alters NR3C1 methylation (epigenetic level), show increased amygdala reactivity to emotional stimuli in early life (neural level). This neural hyperreactivity predicts difficulties in emotional regulation observed during mother–child interactions (relational level), difficulties that may be amplified or attenuated by cultural caregiving practices such as co-sleeping or the availability of alloparental networks (cultural level). The behavioral outcome at 3–5 years of age, which includes differences in emotional regulation, social cognition, and exploratory behavior, thus reflects the dynamic integration of all these domains, rather than the additive contribution of each [21,24,35].

## 2. The First Thousand Days of Life: Neuroplasticity and Environment

The neural plasticity of the fetus and then of the child clearly shows how much researchers and clinicians focus on the environment as a determining factor in the development of the self [36]. The psychoanalytic literature provides us with solid theories on the importance of building the mother–child bond as the basis for this development.

At birth, the newborn is an active and competent subject, with an innate predisposition to relationships. The first interactions with the mother—through gaze, physical contact, and voice—constitute the first affective language, which allows the child to build a sense of continuity and internal coherence. This language is, as already described, implicit (embodied): interaction is contact, gaze, word/sound, movement, and play.

Interaction conveys affection to the child in continuity with the prenatal stages of development. From a psychoanalytic perspective, Winnicott [37] described the concept of “holding” as the maternal function of physical and emotional containment, which allows the child to feel safe and develop trust in the environment. Bion [38], with his theory of the α function, emphasized the importance of the maternal ability to transform raw experiences (β elements) into thinkability, promoting the birth of thought and mind. These qualities of bonding are the basis of healthy child development.

Early interactions are not simple behavioral exchanges, but deeply neurobiological processes that shape brain structure and mentalization capacity [39].

According to Bowlby’s attachment theory [40], children are biologically predisposed to seek the closeness of a caregiver in order to feel protected. Neuroscience confirms that secure attachment is associated with greater integration between the areas of the brain involved in emotional regulation, memory, and stress response. Maternal sensitivity—the ability to perceive, interpret, and respond appropriately to the signals of the newborn—is crucial for the maturation of these circuits. Edward Tronick [41], with his famous ‘still-face’ experiment, demonstrated how a lack of emotional reciprocity, even for short periods, can cause distress in children, highlighting the importance of intersubjective regulation for neurobehavioral development.

One might wonder why the first thousand days of life are the focus of so much attention. This period represents a critical window for brain development. During this period, the brain is characterized by extraordinary synaptic plasticity, which allows for rapid growth and reorganization of neural connections. At no other time do synapse formation and myelination grow so rapidly, after which they undergo a process of remodeling.

From the last trimester of pregnancy, there is an asymmetry in the growth of the hemispheres: the right hemisphere is more developed than the left until the latter catches up in a growth spurt from the second year of life. This means that brain development in this window favors the formation of attachment bonds and affective regulation, both of which are dominated by the right brain.

Early relational experiences, particularly those with the mother figure, modulate the activation of fundamental neurochemical circuits, such as those involving oxytocin, dopamine, and serotonin. These neurotransmitters not only regulate mood and attachment but also influence the maturation of higher cortical structures. This neurochemical maturation helps us understand how even higher cognitive processes, learning, and thinking are the result of more archaic and unconscious emotional learning.

Early affective dysregulation, caused by trauma, neglect, or relational deficiencies, has been associated with compromised neuronal growth, altered stress response, and increased vulnerability to mental disorders in adulthood such as depression, anxiety, dissociative disorders, and relational difficulties. Many syndromes and diagnostic labels currently used in the field of development to classify forms of pathological adaptation take on a deeper meaning thanks to a more complex interpretation, such as that of the intersubjective paradigm.

Antonio Imbasciati has broadened the perspective of child psychoanalysis, shifting the focus from the mind of the individual child to the parent–child relationship as a clinical and theoretical unit. Nonverbal affective communication between mother and newborn constitutes a psychic and neural structure that profoundly affects brain maturation. The early relationship is a structuring psychic situation, capable of producing both psychological and neural effects. Imbasciati’s theoretical framework [42,43,44] redefined the concept of brain maturation, arguing that it depends on primary relational learning and that the parent–child relationship acts as a vehicle for mental transformation through automatic and unconscious affective messages.

## 3. Aim

Building on this gap, the present narrative review pursues three interrelated aims. We first seek to synthesize the empirical and theoretical contributions of four disciplinary domains—psychoanalytic, neuroscientific, genetic and epigenetic, and cross-cultural—that have addressed early attachment in parallel but have rarely been integrated systematically. We then examine the mechanistic interactions between these domains, with particular attention to how biological predispositions, neural plasticity, relational experiences, and cultural practices mutually shape attachment trajectories across the first thousand days of life. On the basis of this integrative synthesis, we propose the Four-Domain Integrative Framework as a heuristic organizational tool for understanding early attachment as a dynamic, co-regulated developmental outcome, and outline its potential implications for clinical practice and future empirical research.

To achieve this aim, we examine three interconnected areas of evidence:

(1) Characteristics of early attachment (Table 2): We identify seven core features of early bonding—proximity seeking, emotional attunement, intrauterine experiences, maternal holding, security patterns, brain plasticity, and maternal stress. Table 2 [8,27,40,45,46,47] describes how these characteristics perform evolutionary functions of protection and adaptation, generating individual differences in emotional, cognitive, and relational development. These characteristics are shaped by genetic/epigenetic mechanisms (polymorphisms in OXTR, 5-HTTLPR, DRD4, and glucocorticoid receptor methylation) that modulate environmental sensitivity [20,21,30,31,33,35]; neurodevelopmental processes (hemispheric specialization, HPA-axis maturation, synaptic plasticity in the first 1000 days) that instantiate attachment at the neural level [8,14,20,21,27,47]; relational experiences (maternal attunement, holding, affect co-regulation) that activate or suppress genetic and neural potentials [37,40,41,46]; and cultural-evolutionary contexts (caregiving ecologies such as co-sleeping, alloparenting, and autonomy promotion) that shape which attachment strategies are adaptive [14,17,18,34].

(2) Cross-cultural variations in caregiving (Table 3): We examine six distinct cultural contexts to demonstrate that secure attachment emerges through culturally specific pathways, differentially influencing motor development, sleep patterns, HPA-axis maturation, and social skills [14,17,26,34,48].

This analysis challenges ethnocentric interpretations and reveals how cultural practices function as scaffolds for gene expression and neural development.

(3) Genetic and epigenetic foundations (Table 4): We analyze five genetic/epigenetic systems that modulate environmental sensitivity according to the differential susceptibility model [19,20,21,33,35,49], demonstrating how biological variability represents an evolutionary hedging strategy that maintains population adaptability.

For each area, we apply the Four-Domain Framework to elucidate mechanistic interactions rather than merely cataloging parallel findings. We show how biological predispositions create windows of differential susceptibility during which neural systems are most plastic and responsive to relational inputs, which are themselves embedded in cultural scaffolds that shape adaptive outcomes. This integrative synthesis carries significant implications for clinical practice, early intervention protocols, and transcultural approaches to perinatal mental health.

### Methodology

This article is a narrative review: although no formal systematic protocol was followed, the search and selection process is described below to allow readers to evaluate the scope and potential limitations of the evidence base. The literature was identified through searches in PubMed, PsycINFO and Google Scholar, covering publications from 1960 to 2024 with no language restrictions. The starting date was chosen to capture the foundational contributions of Bowlby’s attachment theory, the primary theoretical reference point of this review. Search terms included: ‘early attachment,’ ‘mother-infant bonding,’ ‘prenatal attachment,’ ‘first thousand days,’ ‘epigenetics and attachment,’ ‘oxytocin,’ ‘5-HTTLPR,’ ‘differential susceptibility,’ ‘cross-cultural caregiving,’ ‘co-sleeping,’ ‘alloparenting,’ ‘perinatal mental health,’ and ‘neuroscience of attachment.’ Priority was given to peer-reviewed empirical studies with a perinatal or early childhood focus, established theoretical frameworks, and cross-cultural comparative work published in indexed journals; animal studies were included only when providing mechanistic evidence directly complementary to human findings; gray literature, non-peer-reviewed sources, and purely opinion-based contributions were excluded. No formal count of screened versus included sources was maintained, consistent with the narrative nature of the review; selection was guided by theoretical relevance to the Four-Domain Framework and empirical strength of the evidence. Sources were drawn from psychoanalytic, neuroscientific, behavioral genetics, and cross-cultural literatures. Where findings converged across disciplines, this was highlighted as mutually reinforcing evidence; where tensions emerged—for instance, between psychoanalytic constructs and neuroscientific operationalizations of attachment, or between universal claims in attachment theory and cross-cultural variability in caregiving outcomes—these were explicitly flagged and discussed rather than resolved artificially. It should be noted that the sources included in this review vary considerably in terms of the level of evidence they provide. These sources range from randomized controlled trials and longitudinal cohort studies to ethnographic observations, animal models and psychoanalytic theoretical frameworks. Consistent with the narrative nature of this review, no formal quality appraisal was applied. Readers should therefore exercise caution when interpreting the integrative synthesis: the convergence of findings across disciplines is suggestive rather than conclusive, and the Four-Domain Framework proposed here should be understood as a heuristic organizational tool rather than an empirically validated model.

It should further be acknowledged that the literature reviewed does not uniformly support the integrative claims advanced by the Four-Domain Framework. In the domain of gene–environment interactions, the differential susceptibility findings reported by Caspi et al. (2003) [35] for the 5-HTTLPR polymorphism—a cornerstone of the biological domain in this review—have been challenged by subsequent work documenting the complexity and variability of these interaction effects across developmental contexts [50], and by genome-wide studies that have questioned the functional relevance of this variant. Cross-cultural comparisons are similarly constrained by the predominant use of measurement instruments developed and validated in Western contexts, which raises unresolved questions about construct equivalence across caregiving ecologies. In the neuroscientific domain, while associations between early relational quality and neural development are consistently reported, the directionality and specificity of these effects remain difficult to establish given the correlational design and limited sample sizes that characterize much of this literature. These considerations do not invalidate the integrative synthesis proposed here, but they reinforce the characterization of the Four-Domain Framework as a heuristic and organizational tool, whose primary contribution lies in generating theoretically coherent hypotheses for future prospective, multi-level empirical research.

## 4. Cross-Cultural and Genetic Research

Cross-cultural research highlights that, while universal patterns of attachment exist, caregiving practices adapt to specific ecological and social contexts, contributing to different developmental trajectories. Table 3 [14,17,26,34,48] summarizes some of the main alternatives observed in attachment development across cultures. These findings suggest that attachment, even if it is grounded in universal biological foundations, can be expressed through culture-specific pathways with meaningful implications for cognitive, emotional, and social development. Recognizing such variations helps to avoid ethnocentric interpretations and supports the development of clinical intervention protocols and preventive practices that give greater consideration to cultural differences.

Recently, some studies have highlighted the crucial role of genetic foundations and gene–environment interactions in the development of attachment. Specific genetic polymorphisms and epigenetic mechanisms help explain why individuals differ in environmental sensitivity and attachment trajectories. Table 4 [19,20,21,33,35,49] summarizes the main findings.

These findings are consistent with the view that attachment is not only the outcome of relational experiences but rather develops from the dynamic interplay between biological inclinations and environmental contexts. Genetic variability represents an evolutionary strategy that maintains diversity and enables rapid adaptation to changing environments.

From the integrative perspective we propose here, attachment can be understood as the complex outcome of multiple interacting dimensions rather than the product of a single factor.

**Figure 2:**
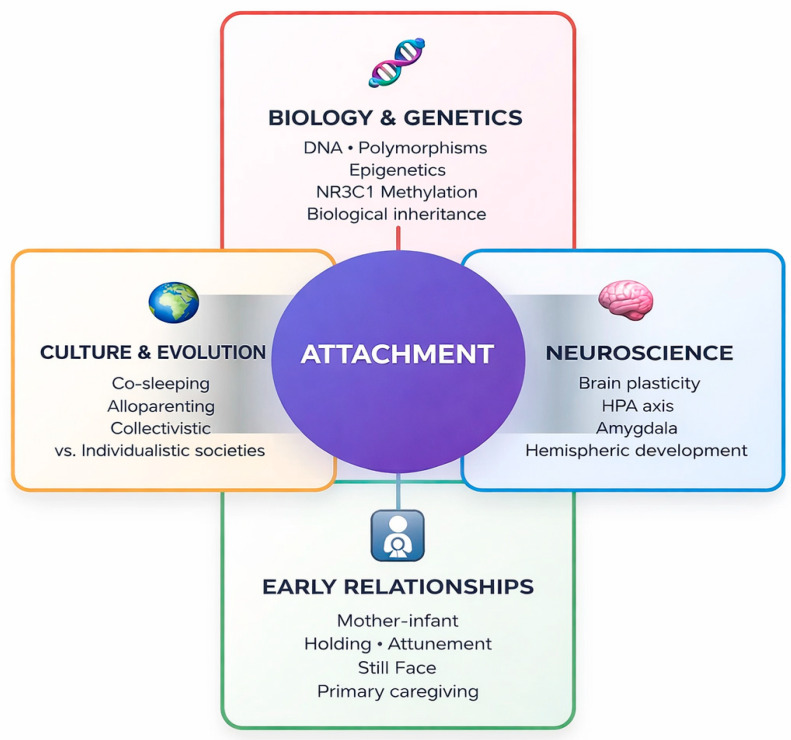
Attachment as a complex result of the interaction between biological/genetic, neuroscientific, relational and cultural factors (adapted from [18,21,41,51,52]). The model illustrates how attachment emerges from the dynamic integration of four fundamental domains: biology (genetic predispositions, polymorphic variants, and epigenetic modifications); neuroscience (brain development, stress systems, and neural plasticity); relationships (quality of early interactions and caregiving patterns); and culture (developmental practices, social contexts, and cross-cultural differences). Each individual develops their own attachment style through the complex and ongoing interaction of these factors.

Contemporary research highlights the converging contributions of biological and genetic mechanisms (e.g., gene polymorphisms and epigenetic processes such as NR3C1 methylation), neuroscientific evidence (brain plasticity, HPA-axis functioning, amygdala activation, hemispheric specialization), early relational experiences (mother–infant attunement, holding, and the Still Face paradigm) and cultural as well as evolutionary contexts (e.g., co-sleeping, alloparenting, and differences between collectivistic and individualistic societies). As summarized in Figure 2, attachment emerges from the dynamic integration of these factors, which together form developmental trajectories and individual differences in socio-relational–emotional functioning [18,21,41,51,52].

## 5. Clinical and Therapeutic Implications

The following considerations reflect theoretical and clinical principles derived from the integrative framework proposed here, rather than direct empirical findings. They are intended as conceptual guidance for practitioners and as hypotheses for future clinical research. Several factors can interfere with the quality of early bonding, including postpartum depression, traumatic experiences, relationship difficulties, and disadvantaged socioeconomic conditions [41,53]. Chronic maternal stress has been associated with alterations in epigenetic transmission and may negatively affect the child’s neurobiological development. In these cases, early intervention by perinatal health professionals is essential to support the mother in her parenting role and promote a supportive relational environment.

Understanding early attachment has profound implications for psychotherapeutic practice in the perinatal period. Perinatal psychotherapy, for example, has shown promising results as a clinical tool for supporting the mother–child dyad, promoting secure attachment and healthy emotional regulation [44]. However, the evidence base for these interventions remains heterogeneous. Interventions with some empirical support include video-feedback programs (e.g., Video-feedback Intervention to promote Positive Parenting, VIPP) and dyadic psychotherapy [28,44]; however, most studies are limited by small samples, a lack of active control conditions, and heterogeneous outcome measures. Large-scale controlled trials remain scarce. Clinical recommendations derived from this literature should therefore be regarded as theoretically informed and preliminary rather than empirically established.

Early intervention, even during pregnancy, may reduce the risk of transgenerational transmission of trauma and promote the child’s harmonious development.

In the clinical setting, it is essential to recognize the signs of early relational distress—such as breastfeeding difficulties, sleep disturbances, irritability—and to intervene with sensitivity and competence [42,54]. The training of practitioners should ideally include neuroscientific knowledge, empathic listening skills, and relational observation tools, though evidence on the effectiveness of specific training formats remains limited.

From this perspective, considering attachment as an adaptive survival function allows us to reinterpret early difficulties not only as indicators of risk, but as defensive strategies that are functional to the context. This may guide therapeutic intervention toward recognizing and transforming these mechanisms into resources for growth and resilience.

Furthermore, the interdisciplinary approach points toward the value of an integrated network of psychologists, pediatricians, midwives, and psychiatrists, a model that remains aspirational in many clinical contexts [55,56,57,58].

The data summarized in the tables are not only of theoretical and evolutionary value but also offer relevant insights for clinical practice. In fact, from a clinical point of view, recognizing that early behaviors (physical contact, attunement, holding) transform into personality structures and styles of affective regulation may allow the therapist to interpret symptoms not as mere deficits, but as adaptive strategies that made sense in a specific relational context. In therapy, this means valuing the patient’s implicit skills and accompanying them in the transformation of rigid strategies into more flexible resources.

In clinical practice, it is essential for the therapist to consider cultural models too and avoid ethnocentric interpretations (e.g., considering an attachment that is functional in another culture to be “insecure”), with the aim to develop a transcultural competence that recognizes the plurality of patterns experienced as secure and safe. In this sense, the therapeutic process does not consist of “normalising” the patient according to Western standards, but rather recognizing and reinforcing attachment patterns that are adaptive in the context to which they belong.

It should be noted that the evidence on genetic polymorphisms and epigenetic modifications opens up clinical perspectives roughly in two directions. The first paves the way for psychoeducational interventions aimed at helping patients (and parents) to understand that vulnerability is not a definitive label, but rather a greater sensitivity to environmental stimuli. On the other hand, the second direction offers opportunities for targeted prevention, namely the awareness that, in genetically more susceptible individuals (“orchids”), early interventions and supportive environments have a particularly transformative effect [19,23]. It should be acknowledged, however, that the translation of these genetic and epigenetic findings into clinical psychoeducation remains largely conceptual at this stage. At present, there are no validated protocols for integrating differential susceptibility profiling into routine perinatal care, and further empirical testing of such approaches is required.

Clinical practice, therefore, is not limited to “curing” but becomes a laboratory of resilience that draws on the plasticity offered by the underlying predispositions.

## 6. Discussion

The synthesis presented in the preceding sections—drawing on psychoanalytic, neuroscientific, genetic, and cross-cultural evidence—provides the empirical and theoretical basis on which the Four-Domain Integrative Framework is proposed. Rather than constituting a pre-formed theoretical lens, the framework emerges from the convergence and tensions identified across these disciplinary domains, and is intended as a heuristic organizational tool for understanding early attachment as a dynamic, co-regulated developmental outcome. In order to facilitate a comprehensive interpretation of the ensuing discourse, it is important to differentiate between three distinct levels of claim: the empirical findings reported in the referenced literature, the theoretical propositions derived from the Four-Domain Integrative Framework, and the clinical implications suggested by the model. Where the evidence is correlational, indirect, or derived from heterogeneous sources, causal language has been deliberately avoided, and statements formulated as hypotheses or implications should not be interpreted as definitive conclusions.

The Four-Domain Integrative Framework we present here proposes to go beyond traditional models by explicating how mechanisms operate across levels simultaneously. Rather than viewing attachment as the sum of independent influences, our model hypothesizes recursive causality: cultural practices may modulate physiological systems, which in turn may shape neural development and influence how children engage with cultural practices. This perspective has practical implications: interventions targeting a single domain (e.g., psychoeducation alone) may be less effective than coordinated approaches that recognize how changes in one domain cascade through others. For example, supporting maternal sensitivity (relational domain) may be enhanced by addressing cultural beliefs about caregiving (cultural domain) while considering individual genetic sensitivity profiles (biological domain) and providing psychoeducation about neural plasticity (neuroscience domain).

As highlighted in the summary in Table 2, basic behaviors (seeking proximity, emotional attunement, intrauterine experiences) are not only immediate adaptive responses, but also matrices of individual differences that endure across the lifespan. It is interesting to note how elements that are apparently “survival” (e.g., physical contact and maternal protection) progressively become complex paradigms linked to trust, motivation, and exploration [59,60,61,62,63]. This pattern is consistent with a hypothesized connection between primary biological needs and long-term psychological trajectories, though direct causal evidence remains limited, demonstrating that the quality of the bond has a deep impact on individual processes of resilience, self-regulation, and psychopathological risk. As shown in Table 2, each characteristic of early attachment operates simultaneously across the four domains of the integrative framework. For example, seeking proximity is not only a relational behavior, but involves the activation of the oxytocin system (biology), modulates amygdala–prefrontal connectivity (neuroscience), responds to maternal sensitivity (relationships), and is calibrated according to cultural norms about appropriate interpersonal distance (culture), demonstrating the recursive causality of the model.

Cross-cultural evidence (Table 3) suggests that, although attachment is universally biological, the ways in which it is expressed and regulated vary according to ecological environments and cultural systems. This broadens clinical reflection: “secure” attachment in Western contexts does not necessarily coincide with the same indicators in collectivist cultures or traditional societies. In this sense, it is important that psychotherapy research takes these cross-cultural peculiarities into account in order to adopt a model that is non-ethnocentric [26,34,64]. Caregiving practices (co-sleeping, alloparenting, reduced social stimulation) show that attachment security can emerge in different ways, all of which are functional to adaptation. Table 3 illustrates how cultural caregiving practices may function as ‘scaffolds’ that simultaneously modulate genetic expression (e.g., OXTR variants), neural development (e.g., cortisol rhythms in co-sleeping), relational patterns (e.g., alloparenting in !Kung) and adaptive outcomes, demonstrating that secure attachment may emerge through culturally specific trajectories rather than following a single universal model.

Individual differences emerge in a complex dynamic and cannot be reduced solely to the environment or relationships, as highlighted in the summary of genetic polymorphisms and epigenetic mechanisms (Table 4). An integrated model emerges in which biology, environment, and culture intertwine and produce different but functional results. Table 4 illustrates how genetic and epigenetic mechanisms are thought not to operate in a deterministic manner, but may create windows of differential susceptibility during which neural systems are most plastic and responsive to relational inputs (e.g., NR3C1 and maternal care), which are in turn embedded in cultural scaffolds (e.g., OXTR variants and collectivist vs. individualistic contexts), exemplifying the cascades of development from the molecular to the behavioral level described in the integrative framework.

In this sense, the variability observed is not an “error” but rather an evolutionary strategy aimed at ensuring flexibility and adaptation. The metaphor of “orchid children” and “dandelion children” becomes particularly powerful in communicating, even at the clinical level, that sensitivity and vulnerability are not deficits but potentials that depend on context [19].

The available evidence, while heterogeneous, suggests the following:

1. Universality and diversity coexist: Although attachment is a universal mechanism, its developmental trajectories differ depending on culture, environment, and genetic predispositions.
2. Prevention and early intervention: Recognizing attachment as an adaptive function implies that even initial difficulties should be interpreted as signs of adaptation to an adverse context. This has clinical implications: early intervention must aim to transform defensive mechanisms into resources for resilience.
3. Transcultural clinical practice: The data highlight the necessity for therapeutic practices that recognize forms of security other than those traditionally accepted in the West.
4. Bio-psycho-social integration: The complexity of early attachment can only be understood through an integrated approach that combines neuroscience, psychoanalysis, and transcultural evidence.
5. Evolutionary perspective: Attachment represents a psychological construct and an evolutionary survival strategy.

The individual’s first emotional experiences are described in the literature on the subject as preparation for social and relational life. From an evolutionary perspective, these experiences constitute adaptive mechanisms which, through the modulation of brain plasticity and emotional regulation [27,47,65], generate individual differences in resilience, motivation, and personality expression.

Selective processes aimed at adaptation to different environmental and social contexts shape the variability observed in the temperaments and developmental trajectories of fetuses and children. Considering attachment as an adaptive function of survival, as well as a psychological construct, allows us to reinterpret the vulnerabilities associated with early bonding dysfunctions as functional mechanisms [65,66,67]: this has significant implications for prevention and clinical practice.

Early emotional bonding is a multidimensional construct that profoundly influences an individual’s mental health, cognitive development, and relational capacity. The integration of neuroscience and psychoanalysis provides a useful theoretical and clinical perspective for understanding the genesis of the mind and promoting preventive therapeutic interventions in the first thousand days of a child’s life.

Contemporary psychotherapy must recognize and value the relational dimension as the foundation of subjectivity. A culture of early emotional care is an investment in future generations’ mental health.

A bio-psycho-socio-evolutionary clinical model that integrates evolutionary, transcultural, and genetic perspectives may offer a useful orientation for the psychotherapist seeking to act as a facilitator of new forms of adaptation [18,21,41,51,52,58].

## 7. Conclusions

The Four-Domain Integrative Framework proposed here suggests that early attachment is best understood as the dynamic, co-regulated outcome of biological predispositions, neural plasticity, relational experience, and cultural context. Several limitations of this review must be acknowledged. The evidence base is heterogeneous, drawing on ethnographic reports, animal models, observational studies, and randomized controlled trials with variable methodological quality. No formal quality appraisal was applied, and a substantial proportion of the sources are conceptual or theoretical in nature. Cross-cultural comparisons are further constrained by small samples and non-equivalent measurement tools. Causal inferences across domains should therefore be interpreted with caution. The Four-Domain Integrative Framework should be understood as a heuristic and integrative model rather than a validated multi-level framework. Its primary contribution is organizational and conceptual, offering a scaffold for future empirical research rather than constituting a confirmed explanatory theory.

## Figures and Tables

**Table 1:** Terminological framework.

Term	Definition	Key Characteristics/Distinctions
**Attachment**	The enduring emotional bond between an infant and a primary caregiver.	Behaviors include proximity seeking, separation protest, and use of the caregiver as a secure base. Patterns may be secure or insecure. Associated with specific neurobiological correlates.
**Bonding**	The process through which the parent (particularly the mother) develops an emotional bond with the newborn, typically in the first hours or days.	Emotional movement from parent to child. Includes hormonal, behavioral, and psychological components. Differs from attachment, which concerns the infant’s bond to the caregiver.
**Early Emotional Bond**	An umbrella term encompassing both attachment and bonding.	Highlights the bidirectional, co-constructed nature of the primary relationship.
**Primary Relationship**	The set of mother–infant interactions and interpersonal dynamics, with references to psychoanalytic constructs.	Includes holding, containment, and alpha function.
**Attunement**	The caregiver’s ability to perceive, interpret, and respond contingently and appropriately to the infant’s emotional states and needs.	Prerequisite for secure attachment. Essential for the development of emotional regulation.
**Usage in the Text**	—	The terms “attachment” and “early emotional bond/bonding” are sometimes used interchangeably when referring to the overall phenomenon addressed by the integrative model.

**Table 2:** Individual differences generated by early attachment.

Characteristic of Early Attachment	Evolutionary Function	Individual Differences Generated	Four-Domain Integration	References
**Proximity Seeking to the Caregiver**	Ensuring protection and survival of the infant in threatening contexts	Levels of secure vs. insecure attachment; predisposition to relational trust	**Biology**: Oxytocin system activation; **Neuroscience**: Amygdala–prefrontal connectivity; **Relationships**: Maternal responsiveness; **Culture**: Acceptable proximity distance varies	[40]
**Mother–Infant Emotional Attunement**	Promoting affect regulation and social cohesion	Differential capacities for self-regulation, resilience, and stress management	**Biology**: Serotonin transporter variants modulate sensitivity; **Neuroscience**: Right hemisphere limbic circuits; **Relationships**: Quality of interactive synchrony; **Culture**: Display rules for emotions	[8]
**Early Intrauterine Experiences (Maternal Voice, Heartbeat, Hormones)**	Preparing the fetus for the postnatal environment and anticipating social interaction	Early temperamental differences (reactivity, sensitivity to stimuli, neurocognitive plasticity)	**Biology**: Epigenetic programming via NR3C1 methylation; **Neuroscience**: Prenatal neural connectivity patterns; **Relationships**: Maternal stress transmission; **Culture**: Pregnancy beliefs affect maternal physiology	[45]
**Maternal Holding and Containment**	Creating a safe environment for the development of the Self	Differences in the development of basic trust and exploratory motivation	**Biology**: HPA-axis calibration; **Neuroscience**: Insula and somatosensory cortex development; **Relationships**: Physical contact frequency; **Culture**: Bodily contact norms (high-contact vs. low-contact cultures)	[46]
**Secure vs. Insecure Attachment**	Fostering cooperation and social adaptation	Divergent trajectories in personality, motivation, and relational capacities	**Biology**: DRD4 variants influence exploration; **Neuroscience**: Prefrontal–limbic integration patterns; **Relationships**: Caregiver sensitivity and availability; **Culture**: Security defined differently (autonomy vs. interdependence)	[8,40]
**Brain Plasticity in the First 1000 Days**	Optimizing adaptation to the environment	Individual variability in memory, attention, and emotional regulation	**Biology**: Synaptic pruning guided by experience; **Neuroscience**: Critical period plasticity mechanisms; **Relationships**: Relational experiences shape neural architecture; **Culture**: Culturally valued skills receive more neural resources	[27,47]
**Early Maternal Trauma or Stress**	Signaling adverse environmental conditions and “preparing” for difficult contexts	Differential vulnerability to anxiety disorders, depression, and affect dysregulation	**Biology**: Glucocorticoid receptor methylation; **Neuroscience**: Altered amygdala reactivity and HPA axis; **Relationships**: Intergenerational transmission patterns; **Culture**: Social support networks buffer or amplify effects	[8,45]

**Table 3:** Cross-cultural variations in attachment development.

Cultural Context	Caregiving Practices	Attachment/Developmental Patterns	Four-Domain Integration	References
**!Kung (Hunter-Gatherers, Botswana)**	- Constant physical contact - Immediate responsiveness - Extended nursing until 3–4 years - Extensive alloparenting	- Rapid motor development - Secure attachment through continuous proximity - Low infant crying	**Biology**: Sustained oxytocin levels from prolonged contact; **Neuroscience**: Enhanced vestibular-motor integration; **Relationships**: Multiple attachment figures (alloparenting); **Culture**: Egalitarian social structure supports shared caregiving	[34]
**Gusii (Kenya, Rural Society)**	- Limited face-to-face interaction - Focus on physical protection and nutrition	- Secure attachment despite different interaction styles	**Biology**: Attachment security achieved via tactile rather than visual modality; **Neuroscience**: Different sensory pathway dominance; **Relationships**: Instrumental care as attachment communication; **Culture**: Agricultural demands shape caregiving priorities	[17]
**Collectivistic Societies (e.g., East Asia)**	- Emphasis on interdependence and emotional control - Group-oriented socialization	- “Culturally modulated secure attachment” - Group social competencies before individual ones	**Biology**: Same oxytocin system, different phenotypic expression; **Neuroscience**: Enhanced social brain network connectivity; **Relationships**: Extended family involvement; **Culture**: Interdependent self-construal shapes secure base function	[48]
**Individualistic Societies (e.g., Western Cultures)**	- Promotion of autonomy and early self-regulation - Valorization of independent exploration	- Attachment patterns oriented toward separation and individuation	**Biology**: Genetic variants (DRD4-7R) may confer advantage in exploration-oriented contexts; **Neuroscience**: Prefrontal systems for self-regulation emphasized; **Relationships**: Dyadic mother–infant focus; **Culture**: Independence valued as developmental milestone	[48]
**Co-Sleeping Practices**	- Shared mother-infant sleep	- Different REM sleep development - Variations in HPA-axis maturation - Differences in nocturnal cortisol levels	**Biology**: Synchronized circadian rhythms; **Neuroscience**: Modified cortisol rhythms affect HPA development; **Relationships**: Continuous nighttime proximity; **Culture**: Co-sleeping as normative practice maintains physiological synchrony	[14]
**Prolonged Carrying Practices**	- Constant infant carrying	- Accelerated vestibular development - Enhanced spatial coordination - Different amygdala activation in response to movement	**Biology**: Increased oxytocin release in both parent and infant; **Neuroscience**: Enhanced vestibular–cerebellar maturation; **Relationships**: Continuous sensorimotor interaction; **Culture**: Babywearing as culturally specific scaffold for development	[26]

**Table 4:** Genetic foundations and gene–environment interactions in attachment.

System/Gene	Key Variants	Effects on Attachment/Development	Four-Domain Integration	References
**Oxytocin Receptor (Oxtr)**	rs53576 (A/G)	**G-allele**: ↑ social sensitivity, secure attachment, better stress regulation; **A-allele**: ↓ social sensitivity, ↑ vulnerability to early trauma; Strong gene × environment effects (caregiving quality)	**Biology**: Genetic variants create differential susceptibility windows; **Neuroscience**: Modulates amygdala-prefrontal coupling; **Relationships**: Effect depends on caregiving quality; **Culture**: Phenotypic expression varies (G-allele more advantageous in collectivist contexts)	[30,31,33]
**Serotonin Transporter (5-Httlpr)**	Short (s) vs. Long (l) allele	**s-allele**: high environmental sensitivity; secure in positive contexts, vulnerable in adverse ones (differential susceptibility model); **l-allele**: low sensitivity; stable development regardless of environment	**Biology**: “Orchid” (s-allele) vs. “dandelion” (l-allele) strategies; **Neuroscience**: s-allele linked to increased amygdala reactivity; **Relationships**: s-carriers benefit more from high-quality caregiving; **Culture**: Prevalence varies across populations, suggesting adaptive polymorphism	[35]
**Dopamine Receptor (Drd4)**	7-repeat variant	Associated with novelty seeking; modulates sensitivity to parenting quality; interacts with disciplinary style → predicts behavioral problems	**Biology**: DRD4-7R confers exploration advantage in novel environments; **Neuroscience**: Affects reward circuitry and executive function; **Relationships**: Interaction with parental warmth vs. harshness; **Culture**: 7R frequency higher in migratory populations, suggesting selection for exploration	[33]
**Epigenetics (NR3C1)**	Glucocorticoid receptor methylation	Maternal stress alters fetal methylation patterns; cross-generational transmission; shapes HPA-axis reactivity lifelong	**Biology**: Epigenetic “memory” of relational context; **Neuroscience**: Programs stress system sensitivity; **Relationships**: Quality of maternal care modulates methylation; **Culture**: Cultural stressors (e.g., discrimination, poverty) transmitted epigenetically	[20,21]
**Fetal Programming Hypothesis**	Prenatal stress vs. protective environment	**Stressful intrauterine conditions**: ↑ reactivity, survival-oriented metabolism; **Supportive conditions**: ↑ regulation, growth, learning, long-term strategy	**Biology**: Predictive adaptive response to expected postnatal environment; **Neuroscience**: Calibrates stress systems prenatally; **Relationships**: Maternal–fetal physiological communication; **Culture**: Societal conditions (war, famine) produce cohort effects	[49]
**Differential Susceptibility Model**	“Orchid” vs. “Dandelion” children	**Orchid**: carriers of sensitivity alleles (5-HTTLPR-s, DRD4-7R); thrive in good contexts, vulnerable in bad ones; **Dandelion**: resilient/stable across environments, less plasticity	**Biology**: Evolutionary hedging strategy maintains population diversity; **Neuroscience**: Susceptibility alleles linked to greater neural plasticity; **Relationships**: Orchids require high-quality caregiving; **Culture**: Different ecologies favor different strategies (stable vs. variable environments)	[19]

↑: increased/higher; ↓: decreased/lower; →: leads to/predicts.

## Data Availability

No new data were created or analyzed in this study. Data sharing is not applicable to this article.
